# Transcriptomic immaturity inducible by neural hyperexcitation is shared by multiple neuropsychiatric disorders

**DOI:** 10.1038/s42003-018-0277-2

**Published:** 2019-01-22

**Authors:** Tomoyuki Murano, Hideo Hagihara, Katsunori Tajinda, Mitsuyuki Matsumoto, Tsuyoshi Miyakawa

**Affiliations:** 1 Division of Systems Medical Science, Institute for Comprehensive Medical Science, Fujita Health University, Toyoake, Aichi 470-1192 Japan; 2 Department of Physiological Science, School of Life Science, SOKENDAI (The Graduate University for Advanced Studies), Kanagawa, 240-0193 Japan; 3 Division of Cell Signaling, National Institute for Physiological Sciences, Okazaki Aichi, 444-8787 Japan; 4 Neuroscience, La Jolla Laboratory, Astellas Research Institute of America LLC, San Diego, CA 92121 USA; 5 Candidate Discovery Science Labs., Drug Discovery Research, Astellas Pharma Inc., Tsukuba 305-8585, Japan

## Abstract

Biomarkers are needed to improve the diagnosis of neuropsychiatric disorders, which are often associated to excitatory/inhibitory imbalances in neural transmission and abnormal maturation. Here, we characterized different disease conditions by mapping changes in the expression patterns of maturation-related genes whose expression was altered by experimental neural hyperexcitation in published studies. This analysis revealed two gene expression patterns: decreases in maturity markers and increases in immaturity markers. These two groups of genes were characterized by the over-representation of genes related to synaptic function and chromosomal modification, respectively. Using these two groups in a transdiagnostic analysis of 87 disease datasets for eight neuropsychiatric disorders and 12 datasets from corresponding animal models, we found that transcriptomic pseudoimmaturity inducible by neural hyperexcitation is shared by multiple neuropsychiatric disorders, such as schizophrenia, Alzheimer disorders, and amyotrophic lateral sclerosis. Our results indicate that this endophenotype serves as a basis for the transdiagnostic characterization of these disorders.

## Abstract (web summary)

Tomoyuki Murano et al. showed that neural hyperexcitation increases the expression of immaturity related genes. These changes in gene expression are shared among different neuropsychiatric and neurological conditions, hinting at their potential role as biomarkers.

## Introduction

Neuropsychiatric disorders—such as schizophrenia, bipolar disorder, major depressive disorder, and autism spectrum disorder—are common, with over a third of the population in most countries being diagnosed with at least one such disorder at some point in their life^1^. Almost all neuropsychiatric disorders are currently classified mainly on the basis of clinical signs and symptoms. However, there is evidence that patients with different clinical diagnoses share similar biological features, such as genetic mutations, molecular expression, and brain activity^2–6^. Recently, psychiatry has undergone a tectonic shift to incorporate the concepts of modern biology. There have been recent attempts to reclassify psychiatric disorders according to biological domains (e.g., genes, neural circuits, and behavior), such as through the Research Domain Criteria (RDoC) initiative^7^. Therefore, identifying appropriate biomarkers that can be used for transdiagnostic assessment of neuropsychiatric disorders is essential for improving the classification of these diseases and understanding their biological basis.

Using coexpression network analysis, a recent study revealed that cross-disorder gene expression overlaps could be used to characterize five major neuropsychiatric disorders^8^. Some of these overlapping gene groups were well characterized biologically by Gene Ontology enrichment or cell-type specificity, but the biological properties of other gene groups were rather unclear. Thus, nonbiased coexpression network analyses do not necessarily detect modules that extract the biological features of neuropsychiatric disorders. Thus, in order to improve the characterization of neuropsychiatric disorders, it might be helpful to detect modules of coexpressed genes and conduct gene expression analysis based on the findings derived from studies on animal models of neuropsychiatric disorders.

To date, we have screened more than 180 strains of genetically engineered mice using a large-scale, and comprehensive battery of behavioral tests, and we have identified several strains with abnormal behaviors related to neuropsychiatric disorders such as schizophrenia, bipolar disorder, and intellectual disability^9^. We discovered common endophenotypes in the brains of multiple strains of these genetically engineered mice with behavioral abnormalities. We termed one such endophenotype in the hippocampus of adult mice the immature dentate gyrus (iDG) phenotype^10–13^. In this phenotype, the molecular and electrophysiological properties of adult DG neurons in the genetically engineered mice were similar to those of immature DG neurons in typically developing infants. For example, the expression of calbindin, a marker of maturity in DG neurons, was decreased, and the expression of calretinin, a marker of immaturity, was increased^10–15^. Molecular changes similar to some of those found in mice with iDG have been observed in the postmortem brains of patients with schizophrenia^16^, bipolar disorder^16^, and epilepsy^17–19^. Furthermore, there is growing evidence that changes in molecular markers of pseudoimmaturity are also present in other brain areas of patients with schizophrenia^20–28^, bipolar disorder^26^, autism^26^, and alcoholism^29^. Therefore, we proposed that pseudoimmaturity of the brain could potentially be a useful transdiagnostic biomarker^9^.

Pseudoimmaturity of the brain can be induced in adulthood. Previously, we found that chronic fluoxetine treatment reversed the maturation status of DG neurons in adult wild-type mice, a phenomenon that we termed dematuration^30,31^. Likewise, recent studies suggest that several maturation-related genes and electrophysiological properties in the DG of wild-type adult mice assume an immature-like status after treatment with pilocarpine or electroconvulsive stimulation^16,32^. As mentioned above, an iDG-like phenotype has been found in patients with epilepsy^17–19^. Therefore, we hypothesized that the neural hyperexcitation may induce pseudoimmaturity of the brain in adulthood; however, this hypothesis has not been tested in human samples.

Some studies suggest that hyperexcitation of neurons may underlie abnormalities related to certain types of neuropsychiatric disorders. Individuals with epilepsy are at increased risk of developing schizophrenia, and vice versa^33,34^; additionally, patients with epilepsy can display psychotic symptoms that resemble those found in patients with schizophrenia^35^. Excitatory/inhibitory imbalances have been proposed to be involved in the pathogenesis and pathophysiology of schizophrenia^36–39^. Hyperactive action-potential firing has also been observed in hippocampal granule-cell-like neurons derived from induced pluripotent stem cells (iPSCs) of patients with bipolar disorder^40^. Recent studies have suggested that human patients with Alzheimer’s disease and temporal lobe epilepsy may harbor common underlying disease mechanisms^17,41–43^. Considering these findings, we hypothesized that the immature-like gene expression patterns induced by neural hyperexcitation may overlap with the abnormal gene expression patterns in the brains of patients with neuropsychiatric disorders and the related animal models. If this is the case, we hypothesized that this overlap can be used to perform transdiagnostic characterization of neuropsychiatric disorders.

To test this hypothesis, we first performed a meta-analysis of microarray datasets, comparing the changes in gene expression in the rat DG after seizure induction with the differences in gene expression in infant mice versus adult mice. To assess consistency across species, we also conducted a similar comparison using the human fetal hippocampus. The overlap between gene sets was estimated using the Running Fisher test^44^, which is a nonparametric statistical method. The fold change and direction of change (up or downregulation) for each gene between the two conditions were used to define the ranked gene signatures. For comparison across different arrays, orthologs were used for each organisms. To determine the similarities between two datasets, we used a combination of rank-based enrichment statistics and ontology-based meta-analysis. This method enables us to statistically assess the pairwise correlations between any two datasets, including datasets from different species and organs^28,29,45,46^. The gene-expression patterns in the rat DG after seizure induction significantly overlapped with those specific to the immature mouse DG and with those specific to the early-stage human fetal hippocampus. From the set of overlapping genes, we defined two groups: maturity-marker genes and immaturity-marker genes that are inducible by neural hyperexcitation. We assessed the expression patterns of these two groups of maturation-related genes in 87 public gene-expression datasets derived from the postmortem brains of patients with various neuropsychiatric disorders and from neural cells derived from patient iPSCs. We further analyzed 12 datasets from the brains of related animal models. Through this analysis, we characterized the expression patterns of maturation-related genes that are inducible by neural hyperexcitation across different disease conditions and animal models of these diseases.

## Results

### Hyperexcitation induces immature-like gene expression

To examine the developmental changes in gene-expression patterns in the rodent DG, we created a microarray dataset from postnatal days 8, 11, 14, 17, 21, 25, and 29 (GSE113727) and compared it with a dataset from 33-week-old adult mice (GSE42778)^12^. Within the entire mouse DG dataset, the largest overlap for changes in gene expression after pilocarpine injection was for the comparison between day 8 infant and 33-week-old adult mice (Supplementary Figure 1a). We included the dataset from postnatal day 8 infant mice for subsequent analysis. The expression levels of 6552 genes were increased in the DG of infant mice compared with adult mice, whereas the expression levels of 8637 genes were decreased (absolute fold change > 1.2 and *t* test *P* < 0.05). Next, we assessed the changes in gene expression induced by neural hyperexcitation in a rodent model. We obtained publicly available microarray datasets from the DG of adult rats after seizures induced by injection of pilocarpine (GSE47752)^47^. The expression levels of 7073 genes were significantly changed in the DG of epileptic-seizure rats 1 day after pilocarpine injection compared with rats treated with saline (absolute fold change > 1.2, *P* < 0.05).

**Fig. 1:**
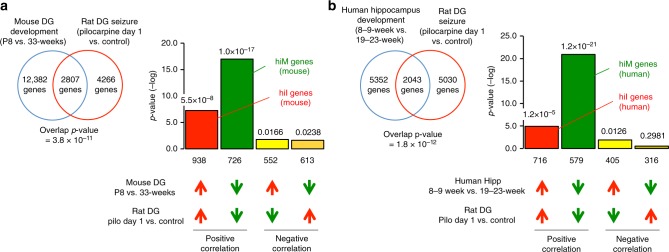
Neural hyperexcitation induces immature-like gene expression patterns in mouse and human. The patterns of changes in gene expression in the rat DG 1 day after pilocarpine treatment compared with developmental changes in the mouse DG and human hippocampus. Venn diagrams illustrating the overlap in genome-wide gene expression changes between the rat DG after seizure induction (GSE47752) and the DG of typically developing mouse infants (GSE113727; P8 infants compared with 33-week-old adults) (**a**) or the hippocampus of typically developing human fetuses (GSE25219: 19- to 23-week fetuses compared with 8- to 9-week fetuses) (**b**). Bar graphs illustrate the −log of the overlap *P* values for genes upregulated (red arrows) or downregulated (green arrows) by each condition. The Bonferroni correction was used to adjust the significance level according to the number of dataset pairs (see the Methods section and Supplementary Methods). Genes that were downregulated in both conditions were defined as mouse hiM genes (green bar in (**a**)), and genes that were upregulated in both conditions were defined as mouse hiI genes (red bar in (**a**)). Similarly, human hiM genes and human hiI genes were defined as the groups of genes with positive correlations between the conditions of development and seizures (**b**)

To investigate whether the neuronal hyperexcitation datasets contain immature-like gene expression patterns, we assessed the overlap between the set of genes with altered expression in immature mice and the set of genes with altered expression in adult seizure-model rats using the Running Fisher algorithm on the BaseSpace platform to determine the significance of the overlap (see Supplementary Methods for details). We found a striking degree of similarity: 2807 genes showed changes in expression in both datasets (overlap *P* = 3.8 × 10^−^^11^) (Fig. 1a). Among these 2807 genes, we named the 726 genes whose expression levels decreased in both datasets hyperexcitation-induced maturity-related genes (hiM genes (mouse): green bar in Fig. 1a) and the 938 genes whose expression levels increased in both datasets hyperexcitation-induced immaturity-related genes (hiI genes (mouse): red bar in Fig. 1a). Comprehensive lists of hiM and hiI genes are in Supplementary Data 1. The overlap for genes with positively correlated expression (red and green bars) was larger than the overlap for genes with negatively correlated expression (light and dark yellow bars), indicating that the directions of expressional change in the two datasets are more often similar than they are different. These results suggest that neuronal hyperexcitation induces a pattern of immature-like gene expression in the adult DG.

Next, we compared the changes in expression during development in the human fetal hippocampus with those in rats after seizure induction to assess consistency across species. We obtained publicly available microarray datasets for the human fetal hippocampus during development (GSE25219)^48^. Within the entire fetal hippocampal dataset, the largest overlap for changes in gene expression after pilocarpine injection was for the comparison between 8- to 9-week fetuses and 19- to 23-week fetuses (Supplementary Figure 1b). Again, we found a striking degree of similarity: 2043 genes showed changes in expression in both datasets (overlap *P* = 1.8 × 10^−12^) (Fig. 1b). Among these 2043 genes, we termed the 579 genes whose expression decreased in both datasets hiM genes (human) (green bar in Fig. 1b) and the 716 genes whose expression increased in both datasets hiI genes (human) (red bar in Fig. 1b). The overlaps for genes with positively correlated expression (red and green bars) were larger than the overlaps for genes with negatively correlated expression (light and dark yellow bars), suggesting that, similar to the results in mice, the gene expression changes in the rat DG after seizure induction are comparable to the reverse of the changes that occur as the human hippocampus develops.

### hiM/hiI genes exhibit different biological properties

To characterize the biological features associated with the hiM and hiI gene groups in mice and humans, we conducted pathway enrichment analyses in BaseSpace. The 20 biogroups that had the largest overlap with the hiM and hiI genes are listed in Tables 1 and 2. Among mouse hiM genes, 4 out of the top 20 biogroups are associated with synapse and channel activity (e.g., transmission of nerve impulse, synapse, and synaptic transmission, Table 1); among human hiM genes, 6 out of the top 20 biogroups are also associated with synapse and channel activity (e.g., transmission of nerve impulse, synaptic transmission, axon, and synapse, Table 2). Among the mouse hiI genes, 4 out of the top 20 biogroups were associated with the nucleus (e.g., genes involved in the cell cycle and genes involved in DNA replication, Table 1). Among the human hiI genes, 15 out of the top 20 biogroups were associated with the nucleus (e.g., genes involved in the cell cycle, chromosomes, and response to DNA damage stimulus, Table 2). Notably, there is little overlap in the top 20 biogroups for the hiM and hiI genes (Table 2). Thus, the biogroups related to the hiM and hiI genes are likely to be functionally different. We further characterized the biological significances of hiM and hiI genes by comparing them with coexpression gene modules obtained from mouse DG development datasets and conducting pathway analyses of those modules. For the detail of the results, see Supplementary Figure 4.

**Table 1:** Summary of results from the pathway analyses of mouse hiM and hiI genes. The 20 biogroups with the most significant similarities to mouse hiM and hiI genes

Biogroup name	Direction	Common genes	*P* value
*hiM genes (mouse)*			
Multicellular organismal signaling	Down	56	4.00E−23
*Transmission of nerve impulse*	*Down*	*51*	*3.10E−19*
Axon	Down	45	7.30E−19
Neuron differentiation	Down	58	4.70E−17
*Synapse*	*Down*	*57*	*5.70E−17*
Neuron development	Down	50	1.50E−16
Neuronal cell body	Down	45	5.20E−16
Cell junction	Down	56	3.00E−15
Cell part morphogenesis	Down	43	4.70E−15
Neuron projection development	Down	42	6.20E−15
Cell projection part	Down	56	6.50E−15
Cell morphogenesis involved in neuron differentiation	Down	35	2.90E−14
Cell morphogenesis involved in differentiation	Down	40	6.70E−14
Cellular chemical homeostasis	Down	53	2.50E−13
Cell–cell signaling	Down	44	2.60E−13
*Synaptic transmission*	*Down*	*37*	*3.60E−13*
Regulation of neurological system process	Down	31	1.10E−12
*Regulation of transmission of nerve impulse*	*Down*	*30*	*1.40E−12*
Genes involved in Neuronal System	Down	31	1.70E−12
Cellular ion homeostasis	Down	49	1.80E−12
*hiI genes (mouse)*			
Response to wounding	Up	60	1.10E−16
Positive regulation of developmental process	Up	66	9.30E−16
Cardiovascular system development	Up	61	7.70E−15
Circulatory system development	Up	61	7.70E−15
Proteinaceous extracellular matrix	Up	32	7.80E−15
**Genes involved in Cell Cycle**	**Up**	**47**	**1.00E−14**
Extracellular matrix	Up	37	1.30E−14
**Genes involved in Cell Cycle, Mitotic**	**Up**	**42**	**6.30E−14**
Protein domain specific binding	Up	60	7.00E−14
Positive regulation of signal transduction	Up	58	8.70E−14
**Genes involved in DNA replication**	**Up**	**32**	**9.20E−14**
**Cell cycle**	**Up**	**67**	**3.00E−13**
Genes involved in adaptive immune system	Up	56	3.20E−13
Focal adhesion	Up	31	5.60E−13
Kinase binding	Up	44	5.50E−12
Cytoskeleton organization	Up	54	5.60E−12
Neuron differentiation	Up	52	5.80E−12
Basement membrane	Up	19	7.50E−12
Regulation of cell migration	Up	36	1.10E−11
MAPKinase Signaling Pathway	Up	20	1.40E−11

Italic columns indicate biogroups that are related to the plasma membrane. Bold columns indicate biogroups that are related to reactions in the nucleus

**Table 2:** Summary of results from the pathway analyses of human hiM and hiI genes. The 20 biogroups with the most significant similarities to human hiM and hiI genes

Biogroup name	Direction	Common genes	*P* value
*hiM genes (human)*			
Multicellular organismal signaling	Up	87	4.40E−58
*Transmission of nerve impulse*	*Up*	*83*	*1.40E−54*
*Synaptic transmission*	*Up*	*75*	*6.70E−50*
Neuron projection	Up	70	2.60E−44
Axon	Up	42	2.90E−33
*Synapse*	*Up*	*51*	*2.60E−32*
Neuron development	Up	58	1.60E−30
Genes involved in neuronal system	Up	41	3.40E−30
*Genes involved in transmission across chemical synapses*	*Up*	*32*	*1.50E−29*
Regulation of neurological system process	Up	35	6.50E−29
*Regulation of transmission of nerve impulse*	*Up*	*34*	*9.20E−29*
Cell projection part	Up	53	1.80E−27
Passive transmembrane transporter activity	Up	44	1.80E−26
*Ion channel activity*	*Up*	*43*	*2.20E−26*
Cell part morphogenesis	Up	49	1.80E−24
Behavior	Up	38	2.90E-24
Single-organism behavior	Up	35	3.70E−24
Cell morphogenesis involved in neuron differentiation	Up	44	4.30E−24
Neuron projection development	Up	47	4.40E−24
Dendrite	Up	37	2.00E−23
*hiI genes (human)*			
**Chromosome**	**Down**	**65**	**1.30E−32**
**Response to DNA damage stimulus**	**Down**	**60**	**4.80E−32**
**Genes involved in Cell Cycle**	**Down**	**50**	**3.60E−31**
**Genes involved in Cell Cycle, mitotic**	**Down**	**43**	**1.20E−30**
**Genes involved in DNA replication**	**Down**	**34**	**2.00E−30**
**Interphase**	**Down**	**47**	**8.20E−30**
**Interphase of mitotic cell cycle**	**Down**	**46**	**5.10E−29**
**Genes involved in mitotic M-M/G1 phases**	**Down**	**29**	**2.60E−26**
**Cell cycle**	**Down**	**25**	**7.60E−23**
**DNA repair**	**Down**	**40**	**2.30E−22**
Wound healing	Down	50	7.40E−22
Response to ionizing radiation	Down	21	1.20E−20
**Nuclear division**	**Down**	**35**	**8.00E−20**
**Mitosis**	**Down**	**35**	**8.00E−20**
**Cell division**	**Down**	**38**	**2.20E−19**
**G1/S transition of mitotic cell cycle**	**Down**	**26**	**1.70E−18**
Cardiovascular system development	Down	46	3.20E−18
Circulatory system development	Down	46	3.20E−18
**S phase**	**Down**	**22**	**3.80E−18**
Blood coagulation	Down	41	6.10E−18

Italic columns indicate biogroups that are related to the plasma membrane. Bold columns indicate biogroups that are related to reactions in the nucleus

We also compared datasets from the DG of typically developing infants with datasets from the rat DG at three different timepoints after seizure induction by injection of pilocarpine or kainate (day 1, day 3, and day 10) and performed principal component analysis on the changes in mouse hiM and hiI genes at different timepoints (Supplementary Figure 2a, b; Supplementary Notes). The time course of changes in the mouse hiM genes after seizure induction was different from the time course of changes in the mouse hiI genes. In addition, we conducted a spatial pattern analysis of the mouse hiM and hiI genes, which indicated that their protein products have slightly different patterns of subcellular localization (Supplementary Figure 2c; Supplementary Notes). The mouse hiM genes tend to be strongly expressed at the plasma membrane, with expression changes stabilizing by the third day after seizure induction. In contrast, the hiI genes tend to be expressed in the nucleus, and changes in expression after seizure induction are slower to stabilize. Together, these results indicate that the hiM and hiI genes have different spatiotemporal patterns of changes in expression.

### Gene-expression analyses of patient samples by hiM/hiI genes

**Fig. 2:**
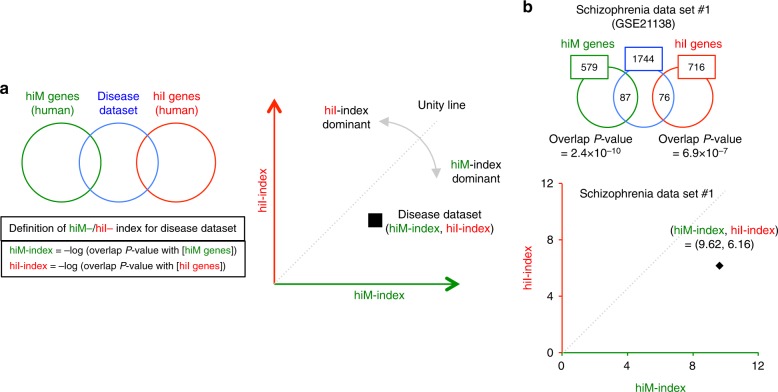
Overview of the two-dimensional (2-D) analysis of disease datasets. **a** Genes with expression changes in the disease datasets are compared with the hiM and hiI gene groups. The hiM- and hiI-indexes were defined as the −log of the overlap *P* values with the hiM and hiI genes, respectively. The gene-expression patterns of the disease datasets are plotted in two-dimensional coordinates, in which the *x*- and *y*-axes are defined by the hiM- and hiI-indexes. Each dataset is characterized as hiM- or hiI-dominant by the ratio of the hiM-index to the hiI-index, and the degree of hiM- or hiI-dominance is evaluated by deviation from the unity line. The distance of each dataset from the origin shows the degree of overlap with hiM-/hiI-genes. **b** 2-D analysis applied to a dataset of postmortem brains (prefrontal cortex) from patients with schizophrenia (schizophrenia dataset #1). The expression levels of 1744 genes are significantly changed in this disease dataset. Of these, 87 and 76 genes overlap with the hiM/hiI genes. The overlap *P* values between the disease dataset and the hiM/hiI genes are 2.4 × 10^−10^ and 6.9 × 10^−7^. The hiM- and hiI-indexes for the disease dataset are therefore 9.62 and 6.16, indicating that this dataset is hiM-dominant. The results of the 2-D analysis for this dataset are plotted in the two-dimensional coordinates defined by the hiM- and hiI-indexes

Next, we investigated whether and to what extent the expression changes in maturation-related genes induced by hyperexcitation overlap with gene expression patterns in various neuropsychiatric disorders. As above, we evaluated similarities between the changes in gene-expression patterns in different groups using overlap *P* values calculated by the Running Fisher algorithm (Fig. 2a). Similarity indexes for each comparison were defined as the −log of the overlap *P* values with hiM or hiI genes, denoted by hiM-index or hiI-index, respectively. High values of the hiM-/hiI-index indicate that there is a large overlap between the dataset analyzed and hiM/hiI genes. We obtained the hiM-/hiI-indexes for the datasets from human patients and plotted them in two-dimensional (2-D) space to show the extent of overlap between datasets and hiM/hiI genes (Fig. 2a).

**Fig. 3:**
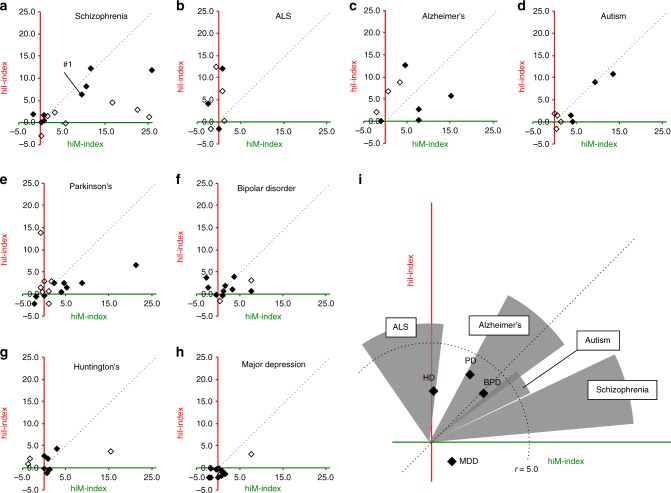
Two-dimensional (2-D) analysis for disease datasets from various neuropsychiatric disorders. **a**–**h** Results of the 2-D analysis of datasets for schizophrenia (**a**), ALS (**b**), Alzheimer’s disease (**c**), autism (**d**), Parkinson’s disease (**e**), bipolar disorder (**f**), Huntington’s disease (**g**), and major depressive disorder (**h**). Each point corresponds to the results of one independent study. Filled points indicate datasets from the postmortem brain or spinal cord (ALS) of patients, and open points indicate those from cultured neural cells from patient iPSCs. **i** The distribution patterns of the hiM- and hiI-indexes for all diseases analyzed. The extent of the changes in hiM-/hiI-indexes is assessed by the average distance of all datasets for each disease from the origin. Four diseases whose average distances from the origin are over 5.0 are shown as circular sectors, and the others are shown as points. The radii of the circular sectors indicate the average distance of all datasets in each disease from the origin, and the central angles of the circular sectors are the average deviation ± SEM from the unity line. Each point indicates the average distance from the origin and average deviation from unity line

We initially performed this 2-D analysis on a dataset containing the expression profile of the prefrontal cortex in the postmortem brains of patients with schizophrenia (schizophrenia dataset #1: details in Supplementary Data 2) (Fig. 2b). The expression of 1744 genes differed between patients and healthy controls (significance level of 0.05). The numbers of hiM and hiI genes with altered expression in schizophrenia dataset #1 were 87 and 76, respectively, and the overlap *P* values were 2.4 × 10^−10^ and 6.9 × 10^−7^, respectively. The hiM-index and hiI-index for this dataset were 9.62 (= −log(2.4 × 10^−10^)) and 6.16 ( = −log(6.9 × 10^−7^)). This result corresponds to a point in 2-D space (Fig. 2b). Points that fall below the unity line (dashed line) indicate datasets in which changes in hiM genes are dominant, whereas points above the unity line indicate datasets with dominant changes in hiI genes. The angle from the unity line indicates the degree of hiM or hiI dominance. The same analysis was performed for other schizophrenia datasets (schizophrenia datasets #2–#16), including those obtained from different areas of the postmortem brain and from cultured neurons derived from the iPSCs of patients. Scatter plots of the results from the schizophrenia datasets are shown in Fig. 3a. Thirteen out of sixteen points were below the unity line. Most of the schizophrenia datasets exhibited hiM-index-dominant patterns, showing high hiM-index values and low hiI-index values (Fig. 3a).

We extended the same analysis to 87 disease datasets from seven other neuropsychiatric diseases (amyotrophic lateral sclerosis (ALS), Alzheimer’s disease (AD), autism spectrum disorder (ASD), Parkinson’s disease (PD), bipolar disorder (BPD), Huntington’s disease (HD), and major depressive disorder (MDD); Supplementary Data 2). The results from each dataset are shown in Fig. 3b–h. The overall distribution patterns of each disease are shown in Fig. 3i. The ALS datasets tended to show higher hiI-index values than hiM-index values, indicating an hiI-index-dominant pattern (Fig. 3b). The AD datasets showed different patterns in the hiM-/hiI-index depending on the type of sample; datasets from the postmortem brains of patients with AD tended to show high values only for the hiM-index, and datasets from patient iPSCs tended to show high values only for the hiI-index (Fig. 3c). Datasets from ASD did not show any dominant patterns in either the hiM-index or the hiI-index (Fig. 3d). Most datasets from patients with PD, BPD, HD, and MDD did not show pronounced values for the hiM-index or the hiI-index (Fig. 3e–h). We applied same analyses on the microarray expression datasets from a recent report by Gandal and colleagues, in which confounding factors are controlled relatively well. Datasets from postmortem brains of patients with schizophrenia and alcohol abuse disorder showed high values for the hiM-index, and those from patients with ASD showed high values for the hiI-index (Supplementary Figure 3).

Thus, the 2-D analysis revealed that some neuropsychiatric diseases have characteristic patterns in the hiM-/hiI-indexes: for example, most datasets from patients with schizophrenia exhibited a higher hiM-index than hiI-index, whereas ALS datasets showed an hiI-index-dominant pattern (Fig. 3i). Meanwhile, different diseases sometimes shared similar changes in the hiM- or hiI-index; for example, some of the schizophrenia, ASD, and AD datasets shared a high hiM-index, and some of the ALS and AD datasets shared a high hiI-index. The other four diseases—PD, BPD, HD, and MDD—did not feature pronounced changes in the hiM-/hiI-indexes, suggesting that these diseases may not share an endophenotype of pseudoimmaturity inducible by neural hyperexcitation. These results raise the possibility that there are patterns of gene expression perturbations that are shared and distinct across these neuropsychiatric disorders.

### Genetic and environmental risks induce pseudoimmaturity

Previous studies suggest that many genetic risk factors and environmental factors, such as seizure, hypoxia, and infection, contribute to the development of neuropsychiatric disorders^2,49,50^. We next applied the 2-D analysis technique to datasets from genetic animal models of disorders and from animals that had experienced risk events.

**Fig. 4:**
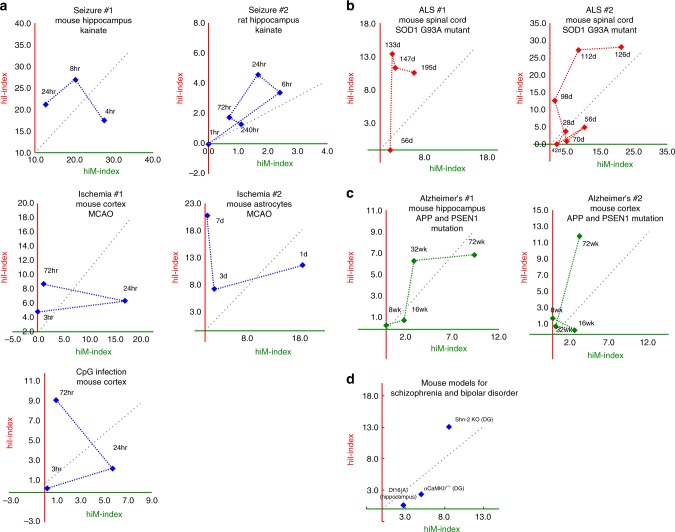
Time-dependent changes in the hiM- and hiI-indexes in animals subjected to various putative risk events for neuropsychiatric disorders and in genetic mouse models of schizophrenia, bipolar disorder, ALS, and Alzheimer’s disease. **a** Pattern of changes in the hiM- and hiI-indexes in the mouse and rat hippocampus after treatment with kainite (seizure #1 (GSE1831) and seizure #2 (GSE4236)), in mouse cortex and astrocytes after middle cerebral artery occlusion (MCAO; ischemia #1 (GSE32529), ischemia #2 (GSE35338)), and in mouse cortex after CpG infection (GSE32529). **b** Pattern of changes in the hiM- and hiI-indexes of the spinal cord of an ALS mouse model with the SOD1(G93A) mutation. **c** Pattern of changes in the hiM- and hiI-indexes of the hippocampus and cortex of an Alzheimer’s disease mouse model with mutations in APP and PSEN1. **d** hiM- and hiI-indexes in mouse models of schizophrenia and bipolar disorder

First, we obtained publicly available datasets for mice that had experienced putative risk events for schizophrenia, bipolar disorders, and Alzheimer’s disease, including seizure (#1: GSE49030, #2: GSE4236)^51,52^, ischemia (#1: GSE32529, #2: GSE35338)^53,54^, and infection (mimicked by CpG; GSE32529)^53,54^. All the studies used here included datasets for different timepoints after the risk event; hence, we were able to examine the time course of changes in the hiM- and hiI-indexes to reveal the short- and long-term effects of risk events on the expression patterns of hiM/hiI genes. The results showed that datasets from the hippocampus of mice treated with kainite, a seizure-inducing drug, exhibited time-course changes in the hiM- and hiI-indexes: the hiM-index tended to be dominant in the early stage after seizure induction, and the hiI-index became more dominant in the late stages (Fig. 4a: seizure #1). The results from other datasets on seizure, ischemia, and infection showed time-course pattern changes in the hiM- and hiI-indexes that roughly matched those observed in seizure dataset #1, being relatively hiM-index-dominant in the early stage and then relatively hiI-index-dominant in the later stage (Fig. 4a: seizure, ischemia, and CpG infection). These results indicate that different types of putative risk events for neuropsychiatric disorders induce roughly similar time-course changes in the expression of maturation-related genes induced by neural hyperexcitation.

Next, we obtained datasets from animal models with a genetic risk of a neurodegenerative disease: mice with transgenic expression of a G93A mutant form of human SOD1, as a model of ALS (#1: GSE46298, #2: GSE18597)^55,56^; transgenic mice with mutant human amyloid precursor protein (APP) and presenilin1 (PSEN1) genes, which cause familial Alzheimer’s disease (#1: GSE64398, #2: GSE64398)^57^; and Df16(A) heterozygous mice carrying a chromosome 16 deletion syntenic to human 22q11.2 microdeletions, as a model of schizophrenia (GSE29767)^58^. We also acquired datasets from Schnurri-2 (Shn-2) knockout mice as a model of schizophrenia^12^ and intellectual disability^14,59,60^ and from mice with heterozygous knockout of the α-isoform of calcium/calmodulin-dependent protein kinase II (α-CaMKII^+/−^)^10,13^ as a model of bipolar disorder^61^. These mice show an array of behavioral abnormalities, including locomotor hyperactivity and severe deficits in working memory. The expression profiles of several maturation marker genes in exhibit immature-like patterns in these mice^10,12^. The threshold current of granule cells to induce spikes was low in these mice, indicating that the granule cells of these mice are highly excitable^10,12^. We performed 2-D analysis on these datasets and evaluated the changes in the hiM-/hiI-indexes of these model mice. Both datasets from transgenic mice with the SOD1(G93A) mutation exhibited an hiI-index higher than the hiM-index in the later stages of disease progression (Fig. 4b). These hiI-index-dominant patterns were also observed in the results derived from human patients with ALS (Fig. 3b). In the mice with mutant human APP and PSEN1, both the hiM- and hiI-indexes increased in the dataset from the hippocampus, and only the hiI-index increased in the dataset from the cortex during the course of disease progression (Fig. 4c). These patterns are neutral or hiI-index dominant and partially mimic the results from human patients with Alzheimer’s disease (Fig. 3c). The Df16(A) heterozygous mice and α-CaMKII^+/^^−^ mice showed hiM-index-dominant patterns, similar to results from human patients with schizophrenia (Fig. 4d). Shn-2 KO mice showed high values for both the hiM- and hiI-indexes (Fig. 4d). Thus, the results from the 2-D analysis of animal models are to some extent consistent with the results from human patients, indicating that these model mice have patterns of pseudoimmaturity induced by neural hyperexcitation that are similar to those in human patients. Potential confounding factors, such as types of samples, brain areas, and age, were less numerous in the animal-model analyses than in the analyses of samples from human patients, establishing the causal links between experimental manipulations corresponding to disease conditions and pseudoimmaturity inducible by hyperexcitation.

## Discussion

In this study, we demonstrated that neural hyperexcitation induces changes in the pattern of gene expression in the DG that are similar to the patterns in the immature hippocampus of typically developing human fetuses. From the pool of genes, we identified two groups of genes, and found that these are shared by multiple neuropsychiatric disorders, such as schizophrenia, Alzheimer disorders, and ALS.

Many of the datasets from patients with schizophrenia and from the postmortem brains of patients with Alzheimer’s disease exhibited hiM-index-dominant pattern changes. The hiM genes, include genes encoding a GABA receptor, voltage-dependent calcium channel, glutamate receptor, and voltage-dependent sodium channel (Supplementary Data 1). These genes have been implicated in the pathological changes in the brains of patients with schizophrenia and Alzheimer’s disease^62–65^. Thus, many of the synaptic genes that changed in the brains of patients with schizophrenia or Alzheimer’s disease could be genes whose expression increases during maturation and decreases with neural hyperexcitation. Although reductions in the expression of some synaptic genes in these disorders are well documented, to our knowledge, our results are the first to raise the possibility that neuronal hyperexcitation may also induce reductions in such synaptic molecules. Most of the datasets from patients with ALS and Alzheimer’s disease exhibited hiI-index-dominant patterns. The hiI genes, include genes encoding DNA methyltransferase, cyclin D1, cyclin-dependent kinase 1, integrin beta 3 binding protein, and tumor protein p53 (Supplementary Data 1). These genes are known to be important in chromosomal modification and DNA repair, and abnormal functioning of these systems has been observed in patients with ALS and Alzheimer’s disease^66–70^. Thus, some of the genes that are considered important in the development of these disorders are immaturity-related genes, whose expression decreases during maturation and can be increased by neural hyperexcitation. As for the datasets from patients with PD, BPD, HD, and MDD, most of them did not overlap with either hiM or hiI genes, suggesting that there might not be major pathological changes in transcriptomic pseudoimmaturity inducible by neural hyperexcitation in the datasets of these four diseases. Thus, we were able to characterize the gene expression patterns in disease datasets of each disease category using the hiI- and hiM-indexes.

Our study has some limitations. First, the number of available datasets was limited. All the datasets except the one for mouse development were obtained from the BaseSpace Correlation Engine. On this platform, vast numbers (over 21,000) of complex biological and clinical datasets are available. Although we used all the gene expression dataset hits from our keyword query to avoid sampling bias, the number of datasets was still small, from 8 datasets for ALS to 16 datasets for schizophrenia. Further accumulation of the studies will improve the reliability of our results. Another limitation is that the datasets used in this study are from different types of samples, including various areas within the central nervous system, such as the hippocampus, prefrontal cortex, striatum, and spinal cord. The gene expression abnormalities in patients could differ depending on the brain area^48^. We also used datasets from cultured neurons differentiated from the iPSCs of human subjects, and it is controversial whether the pattern of gene expression in these neurons is comparable to that of neurons in the patients’ brains^71,72^. It is also possible that the altered gene expression in the postmortem brains is due to the effects of medication rather than pathological changes from the disease itself^73^. We compared the expression patterns of the genes derived from rodents and humans. There could be critical differences among different species, which might have caused the analysis to miss important molecular regulatory elements shared among those neuropsychiatric disorders. Other conditions that were not controlled in this study and can be potential confounding factors include the age of subjects at death, the storage conditions of the samples, the genetic background of animals, and the animal housing conditions. For these reasons, we need to be careful in interpreting the results of the analyses. It should be noted, however, that despite the variety of sample types used, we were able to identify some shared and distinct patterns of gene expression, which is mostly due to the advantages of utilizing the Running Fisher test; this test, using a rank-based nonparametric algorithm, can evaluate similarity between datasets from different species or organs^44^. Moreover, we applied 2-D analysis to the animal models, in which potential confounding factors such as species, sample types, and brain areas are well controlled. The results from these models were comparable to those from human samples, supporting the idea that pseudoimmaturity inducible by hyperexcitation is a feature shared by multiple neuropsychiatric disorders. It should be noted, however, that a gene expression pattern resembling those induced by neural hyperexcitation may not be induced by neural hyperexcitation per se but by other factors such as inflammation, hypoxia, and infection. Another potential advantage of using the Running Fisher test is that this method is applicable to the dataset of gene expression profiles from an individual patient or control. In the future, it might be interesting to apply this method to the characterization and classification of gene expression patterns in the brains of individual patients and in neurons derived from patients’ iPSCs.

Recent attempts such as the RDoC initiative have tried to reclassify psychiatric disorders according to biological domains (e.g., genes, neural circuits, and behavior)^7^. While Gandal et al.^8^ conducted nonbiased coexpression analyses, this study utilized gene groups derived from the findings based on the studies of animal models of neuropsychiatric disorders. Characterization by these gene groups enabled us to extract novel biological features of some neuropsychiatric disorders that are related to pseudoimmaturity inducible by neural hyperexcitation. Detecting such domains that extract the biological features of each neuropsychiatric disorder will move this diagnostic framework forward, from criteria based on signs and symptoms to those including biological dimensions.

In conclusion, the biological domain of pseudoimmaturity inducible by neural hyperexcitation is a common endophenotype among several neuropsychiatric disorders. Future studies are needed to find translational indices that correspond to these features and can be applicable to human patients for better diagnosis of these neuropsychiatric disorders. Our findings here may promote the development of biomarkers, leading to a better diagnosis of neuropsychiatric disorders.

## Methods

### Microarray experiments to examine mouse DG development

Wild-type mouse DGs were sampled at postnatal days 8, 11, 14, 17, 21, 25, 29 (C57BL/6J × BALB/cA background; male, *n* = 5)^74^, and microarray experiments were performed with Mouse Genome 430 2.0 Array (Affymetrix, Santa Clara, CA) as previously described^10^. RNA was isolated by using the TRIzol method (Invitrogen, Carlsbad, CA) from the hippocampus of mice, followed by purification, using RNeasy columns (Qiagen, Valencia, CA). Double-stranded cDNA was synthesized from the total RNA, and invitro transcription reaction was then performed on biotin-labeled RNA that was made from the cDNA. Labeled RNA was hybridized with Mouse Genome 430 2.0 Array (Affymetrix, Santa Clara, CA) containing 45,101 probe sets, and washed according to the manufacturer’s recommendations. The hybridized probe array was then stained with streptavidin-conjugated phycoerythrin, and each GeneChip was scanned by an Affymetrix GeneChip Scanner 3000 (GCS3000). GeneChip analysis was performed with Microarray Analysis Suite version 5.0. All of the gene represented on the GeneChip were globally normalized. Genes with a *P* value < 0.05 (without correction for multiple testing) and an absolute fold change >1.2 were included in the differentially expressed gene datasets. The microarray data, including those used in this study, were deposited in the Gene Expression Omnibus (GEO) database under accession number GSE113727. We also obtained a dataset for 33-week-old wild-type mice, which we previously reported (C57BL/6J × BALB/cA background) (GSE42778)^12^. We integrated these two datasets into one to construct the dataset for the development of the wild-type mouse DG used in this study (P8 versus adult, fold change >1.2, *P* < 0.05). All animal experiments were approved by the Institutional Animal Care and Use Committee of Fujita Health University, based on the Law for the Humane Treatment and Management of Animals and the Standards Relating to the Care and Management of Laboratory Animals and Relief of Pain. Every effort was made to minimize the number of animals used.

### Data collection and processing

Except for the mouse DG developmental dataset mentioned above, the 99 gene expression datasets used in this study were obtained from publicly available databases (listed in Supplementary Data 2). We used all the hits from our keyword query for gene-expression studies to avoid sampling bias. If a single study included several different datasets, we chose the one that we considered the most comprehensive (e.g., if the study includes datasets from male, female, and all subjects, we used datasets from all subjects). All gene-expression datasets were analyzed with the BaseSpace Correlation Engine (formerly known as NextBio) (https://japan.ussc.informatics.illumina.com/c/nextbio.nb; Illumina, Cupertino, CA), a database of biomedical experiments. BaseSpace is a repository of analyzed gene-expression datasets that allows researchers to search expression profiles and other results^44^. The datasets registered in BaseSpace undergo several preprocessing, quality control, and organization stages. Quality control ensures the integrity of the samples and datasets and includes evaluations of prenormalization and postnormalization boxplots, missing value counts, and *P* value histograms (after statistical testing) with false-discovery rate analysis to establish whether the number of significantly altered genes is larger than that expected by chance.

Genes with a *P* value < 0.05 (without correction for multiple testing) and an absolute fold change >1.2 were included in the differentially expressed gene datasets. This sensitivity threshold is typically the lowest used with commercial microarray platforms and the default criterion in BaseSpace analyses^44^. Correction for multiple testing was omitted to minimize false negatives at this stage. All data from the Affymetrix GeneChip series were downloaded from the NCBI GEO database. Affymetrix Expression Console software (specifically, the robust multiarray average algorithm) was used to preprocess the data.

We used the expression values (on a log base 2 scale) to calculate the fold changes and *P* values between two conditions (infants–adults and patients–healthy controls). To determine the fold changes, we began with the expression values of the probes/genes in the test data sets and divided them by those of the control data sets. If the fold change was <1.0, these values were converted to the negative reciprocal, or −1/(fold change). Genes with an absolute fold change >1.2 and a *t* test *P* value < 0.05 were imported into BaseSpace Correlation Engine according to the instructions provided by the manufacturer. The rank order of these genes was determined by their absolute fold change. All statistical analyses were performed in BaseSpace, and the similarities between any two datasets were evaluated as overlap *P* values using the Running Fisher algorithm^44^. The Bonferroni correction was used to adjust the significance level according to the number of datasets pairs^44^.

## Supplementary information

Supplementary Information

Description of Additional Supplementary Files

Supplementary Data 1

Supplementary Data 2

Supplementary Data 3

Supplementary Data 4

Supplementary Data 5

Supplementary Data 6

Supplementary Data 7

Supplementary Data 8

Supplementary Data 9

Supplementary Data 10

Supplementary Data 11

Supplementary Data 12

## Data Availability

The microarray and RNAseq data that support the findings of this study have been available in the BaseSpace Correlation Engine (https://japan.ussc.informatics.illumina.com/c/nextbio.nb; Illumina, Cupertino, CA) and Gene Expression Omnibus (GEO; https://www.ncbi.nlm.nih.gov/geo/). All detailed information, including accession numbers, is listed in Supplementary Data 2.
