# Author Correction: Transcriptomic immaturity inducible by neural hyperexcitation is shared by multiple neuropsychiatric disorders

**DOI:** 10.1038/s42003-019-0358-x

**Published:** 2019-03-04

**Authors:** Tomoyuki Murano, Hideo Hagihara, Katsunori Tajinda, Mitsuyuki Matsumoto, Tsuyoshi Miyakawa

**Affiliations:** 1 Division of Systems Medical Science, Institute for Comprehensive Medical Science, Fujita Health University, Toyoake, Aichi 470-1192 Japan; 2 Department of Physiological Science, School of Life Science, SOKENDAI (The Graduate University for Advanced Studies), Kanagawa, 240-0193 Japan; 3 Division of Cell Signaling, National Institute for Physiological Sciences, Okazaki Aichi, 444-8787 Japan; 4 Neuroscience, La Jolla Laboratory, Astellas Research Institute of America LLC, San Diego, CA 92121 USA; 5 Candidate Discovery Science Labs., Drug Discovery Research, Astellas Pharma Inc, Tsukuba, 305-8585 Japan

Correction to: *Communications Biology* 10.1038/s42003-018-0277-2; Published online 22 January 2019

In the original published version of this article, Supplementary Data 1 reported gene expression fold changes incorrectly. The original supplementary file reported all fold changes as raw values, whereas the main text explains that genes with fold changes <1 were converted to the negative reciprocal (−1/(fold change)). This error has been corrected in the Supplementary Data 1 file.

