# Supplementary material for: Transcriptomic immaturity inducible by neural hyperexcitation is shared by multiple neuropsychiatric disorders
Source: Commun Biol. 2019 Jan 22;2:32. doi: 10.1038/s42003-018-0277-2 (PMC6342824; doi:10.1038/s42003-018-0277-2)
Supplement: Supplementary file 1 — Supplementary Information [file 42003_2018_277_MOESM1_ESM.pdf]

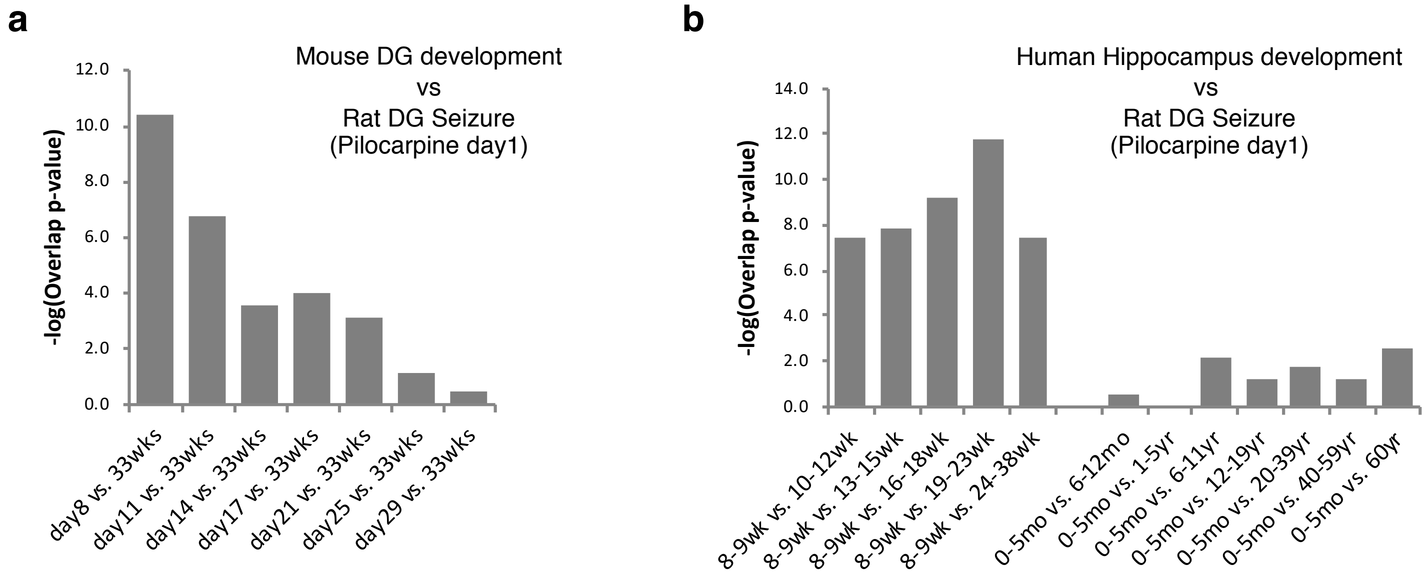

Supplementary Figure 1.

The pattern of changes in gene expression in rat DG 1 day after pilocarpine treatment compared with various developmental stages in mouse DG (a) and human hippocampus (b). Gene expression changes in rat DG after seizure induction (GSE47752) and the DG of developing mice at various stages (GSE113727, infants: P8, P11, P14, P17, P21, P25, P29; adults: 33 weeks) (a) or the hippocampus of typically developing human fetuses (GSE25219: 10–12-week, 13–15-week, 16–18-week, 19–23-week, and 24–38-week fetuses compared with 8–9 week fetuses, or 6–12-month, 1–5-year, 6–11-year, 12–19-year, 20–39-year, 40–59-year, and 60+-year adults compared with 0–5-month infants) (b). Bar graphs illustrate the −log of overlap *P*-values between conditions. The dataset from the DG of day 8 infant mice versus 33-week adults had the largest overlap with the dataset for rat DG 1 day after seizure induction versus controls. The dataset from the hippocampus of 19–23-week fetuses versus 8–9-week fetuses showed the largest overlap with the dataset for rat DG 1 day after seizure induction versus controls.

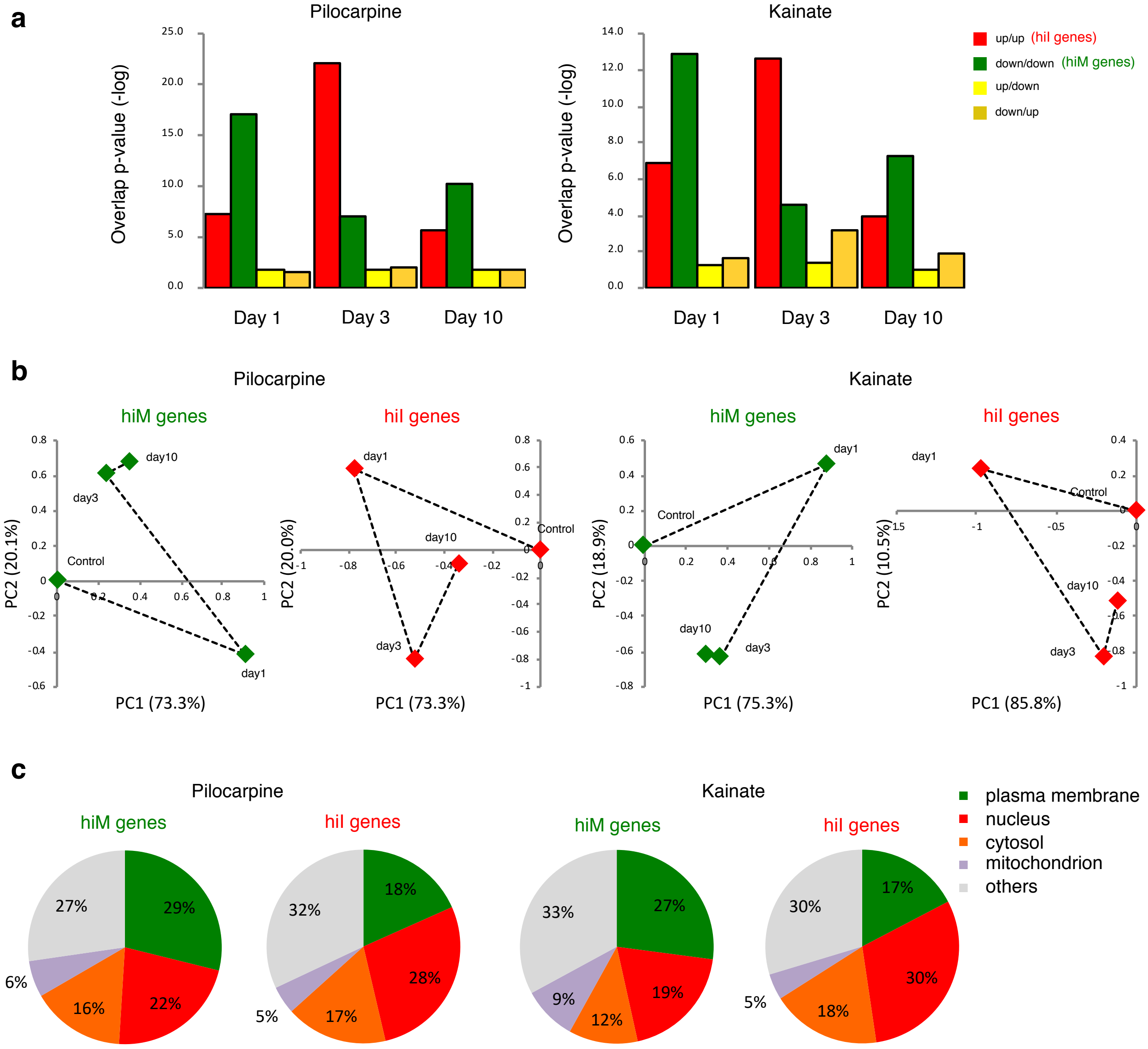

Supplementary Figure 2.

Differences in the spatiotemporal patterns of the hyperexcitation-induced expression changes in hiM and hiI genes. (a) Gene expression changes in rat DG at three time points after seizure induction (pilocarpine or kainite) compared with the differences between infant and adult mouse DG. Bar graphs illustrate the overlap *P*-values for genes upregulated or down-regulated in each condition. The Bonferroni correction was used to adjust the significance level according to the number of dataset pairs. (b) The results of principal component analysis for the time-course expression pattern changes in hiM and hiI genes after seizure induction. (b) Subcellular distribution patterns of hiM and hiI genes. Pie charts show the subcellular distribution ratios of the 50 genes with the highest fold change included in each gene set.

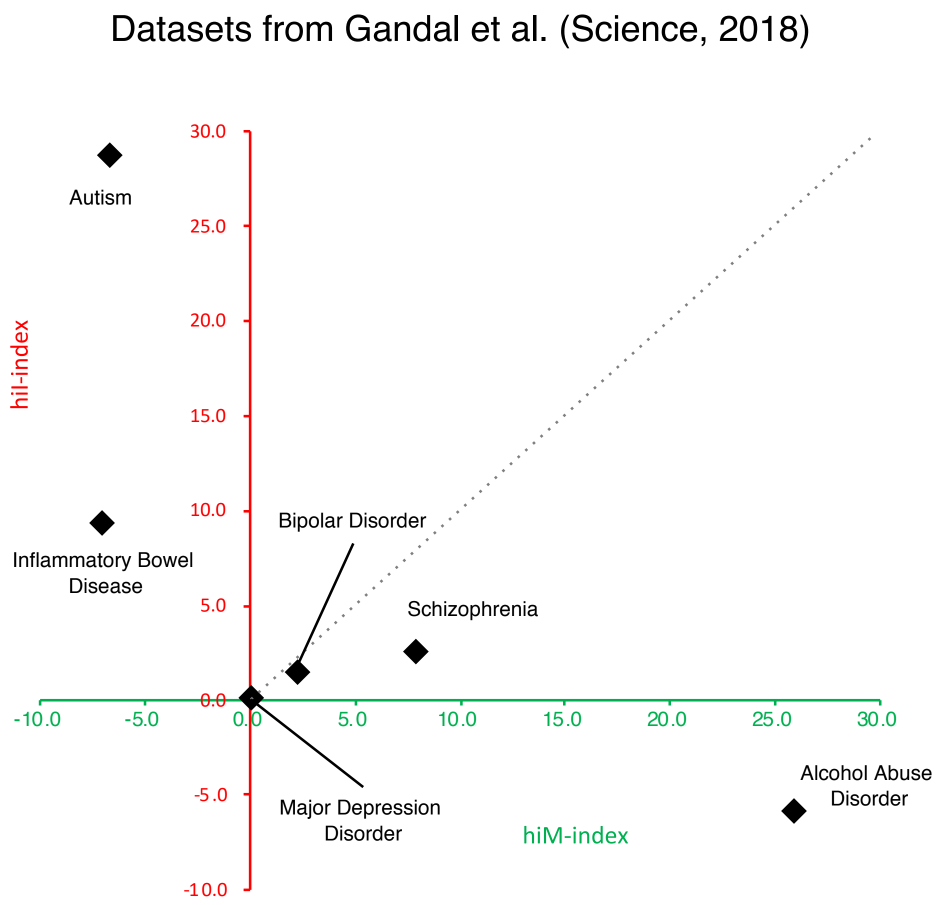

Supplementary Figure 3.

Results of 2-D analysis performed on the datasets from cerebral cortical samples of subjects with major neuropsychiatric disorders (Gandal et al., Science, 2018). Each dot corresponds to results of microarray datasets from each disease. hiM-index-dominant pattern were found in schizophrenia and bipolar disorder/major depression disorder show small changes in both of hiM and hiI index, which is mostly consistent with our results in Figure 3. Autism show changes in hiI index but not in hiM index, which is different pattern obtained in our results. Alcohol abuse disorder and inflammatory bowel disorders, which were not assessed in Figure 3, show hiM-index-dominant and hiI-index-dominant patterns.

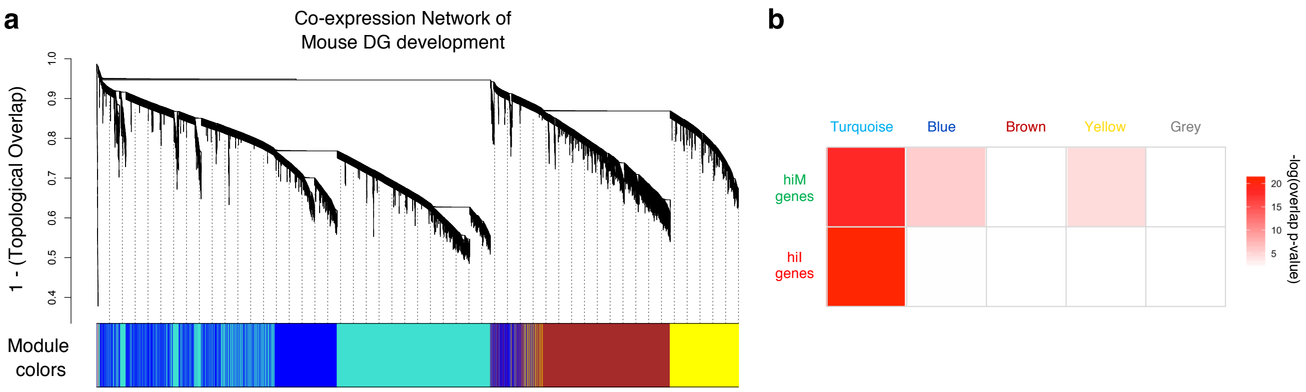

c

Module: Turquoise (7649 genes)		
**Biogroup name**	**common genes**	**p-value**
cell division	201	2.60E-39
Genes involved in Cell Cycle	179	1.20E-30
mitosis	144	6.30E-30
Genes involved in Cell Cycle, Mitotic	155	3.60E-29
Genes involved in Developmental Biology	167	1.50E-23
positive regulation of cell differentiation	196	4.10E-23
Genes involved in Axon guidance	120	1.20E-22
Genes involved in DNA Replication	101	1.30E-21
brain development	161	5.40E-21
negative regulation of cellular component organization	131	5.90E-21

Module: Blue (5424 genes)		
**Biogroup name**	**common genes**	**p-value**
GTPase regulator activity	120	3.70E-11
dendrite	127	2.10E-09
dendritic spine	62	5.00E-07
regulation of small GTPase mediated signal transduction	91	7.30E-07
synapse	149	9.20E-07
regulation of anti-apoptosis	25	1.30E-06
neuronal cell body	118	1.50E-06
Genes involved in Neuronal System	76	2.50E-06
Genes involved in Transmission across Chemical Synapses	57	3.00E-06
enzyme activator activity	92	7.50E-06

Module: Brown (4568 genes)		
**Biogroup name**	**common genes**	**p-value**
Large Drosha complex	9	0.004
cytoplasmic sequestering of protein	7	0.0045
gene silencing	16	0.0109
transcription elongation factor complex	9	0.0178
mitotic spindle	6	0.0182
Genes involved in Transcriptional activity of SMAD2/SMAD3:SMAD4 heterotrimer	10	0.0197
Genes involved in Signaling by TGF-beta Receptor Complex	16	0.0203
vacuolar transport	11	0.0219
lysosomal transport	10	0.0358
G alpha q Pathway	9	0.0373

Module: Yellow (2454 genes)		
**Biogroup name**	**common genes**	**p-value**
perinuclear region of cytoplasm	64	6.80E-06
sarcolemma	22	1.00E-05
Genes involved in Developmental Biology	60	5.50E-05
protein N-terminus binding	22	5.70E-05
Golgi-associated vesicle membrane	9	0.0001
mitochondrial membrane	63	0.0002
Genes involved in NGF signalling via TRKA from the plasma membrane	28	0.0003
Genes involved in Keratan sulfate/keratin metabolism	9	0.0003
cell-substrate adhesion	21	0.0003
14-3-3 protein binding	7	0.0004

Module: Grey (76 genes)		
**Biogroup name**	**common genes**	**p-value**
cell surface receptor signaling pathway involved in heart development	2	0.0008
regulation of transcription regulatory region DNA binding	2	0.0013
Beta1 integrin cell surface interactions	3	0.0013
pattern specification process	5	0.0019
cellular lipid catabolic process	3	0.0023
negative regulation of leukocyte apoptotic process	2	0.0023
Genes involved in Nuclear signaling by ERBB4	2	0.0029
Genes involved in Tight junction interactions	2	0.0031
Genes involved in Amyloids	2	0.0036
BMP receptor signaling	2	0.004

Supplementary Figure 4.

Comparison between gene modules obtained from mouse DG development datasets and hiM/hiI genes. (a) Network dendrogram from coexpression topological overlap of genes across disorders. (b) Overlap between genes in hiM/hiI genes and 5 gene modules obtained from coexpression analyses in (a). Color bars show -log of overlap *P*-value. (c) Summary of results from the pathway analyses of 5 modules.

Supplementary Notes

To evaluate the time-course of changes in immature-like gene expression patterns after seizure induction, we compared datasets from the DG of typically developing infants with datasets from rat DG at three different timepoints after seizure induction by injection of pilocarpine or kainite (day 1, day 3, and day 10). As shown in Supplementary Figure 2a, the overlap *P*-values for the hiM genes were smaller than those for the hiI genes on the first day after seizure induction in both the pilocarpine and kainate datasets. Interestingly, the overlap *P*-values for the hiI genes were smaller than those for the hiM genes on day 3. These differences in the overlap *P*-values of the hiM/hiI genes with the number of days after seizure induction suggest that the expression levels of maturity-/immaturity-related genes induced by neural hyperexcitation change over time; the expression changes of hiM genes in the DG occur earlier than those of hiI genes after seizure induction. The similarity of the results for pilocarpine and kainite also suggests that the induction of immature-like gene expression is not dependent on the specific seizure-inducing drugs but on the induced neural hyperexcitation itself.

We performed principal component analysis (PCA) to visualize the relationships between hiM/hiI genes at different timepoints after seizure induction. For these analyses, we generated integrated datasets comprised of hiM or hiI genes at three different timepoints after seizure induction by pilocarpine or kainite (pilocarpine hiM gene sets, pilocarpine hiI gene sets, kainite hiM gene sets, and kainite hiI gene sets) and performed PCA for each dataset. Datasets at every timepoint after seizure induction and those of untreated control were plotted in a two-dimensional space with coordinates corresponding to principal components (PC) 1 and 2 (Supplementary Figure 2b). The PC1/PC2 coordinates of the hiM genes are different for day 1, day 3, and the untreated control, but those for day 3 and day 10 are close to each other (Supplementary Figure 2b), indicating that the expression changes in hiM genes after seizure induction become stable by day 3. However, the PC coordinates for hiI genes differed from each other at all timepoints after seizure (Supplementary Figure 2b), indicating that the expression changes in hiI genes continue after day 3 until at least day 10. These results also suggest that the changes in expression of the hiM genes follow a different temporal pattern than the changes in expression of hiI genes.

We conducted a bioinformatic estimation of the subcellular distributions of the protein products of hiM/hiI genes. To predict their subcellular distribution, we used the open-source tool COMPARTMENTS, which provides information on the estimated subcellular localization of genes of interest (see above). The subcellular distributions of

hiM genes in the plasma membrane/nucleus were 28%/16% (in the pilocarpine dataset) and 26%/15% (kainite), and those of hiI genes were 16%/20% (pilocarpine) and 16%/23% (kainate) (Supplementary Figure 1c). The proportion of expression in the plasma membrane was higher in the hiM gene group than in the hiI gene group, while that in the nucleus was higher in the hiI gene group than in the hiM gene group. These results indicate that the protein products of hiM/hiI genes tend to have spatially distinct localizations.

We applied our analysis on the microarray expression datasets generated by Gandal and colleagues, in which confounding factors are largely controlled (Supplementary Figure 3). The hiM-index and hiI-index of datasets from patients with schizophrenia were 7.85 and 2.46, respectively, indicating hiM-index-dominant pattern. This result indicates that schizophrenia datasets used in Gandal's study also show transcriptomic pseudoimmaturity inducible by neural hyperexcitation. The hiM-index and hiI-index of datasets from bipolar disorder patients were 2.19 and 1.48, respectively, and those of major depressive disorder were both 0. These indexes are smaller than those of schizophrenia, which is mostly consistent with our results in Figure 3. The hiM-index and hiI-index of autism were -6.74 and 28.66, respectively. This hiI-index-dominant pattern is different from our results, as shown in Figure 3d and 3i. Although the pattern in autism is apparently different from ours, datasets from autism show significant overlap with pseudoimmaturity inducible by hyperexcitation, regarding hiI-index. We have added analysis of the datasets from alcohol abuse disorder and inflammatory bowel disease. Their hiM-/hiI-indexes were 25.85/-5.92 and -7.03/9.31, respectively.

To further evaluate biological significances of hiM and hiI genes, we performed weighted gene coexpression network analysis (WGCNA), which is a common method in systems biology for describing the correlation patterns among genes across microarray samples based on the k-means algorithms. We applied this method to the datasets from mouse DG development (postnatal day 8, 11, 14, 17, 21, 25, and 29 mouse infants and 33-week-old adult mice), and extracted 5 modules (turquoise, blue, brown, yellow, and grey) (Supplementary Figure 4a). We compared hiM and hiI gene groups with these 5 modules and evaluated their similarities by Running Fisher test (Supplementary Figure 4b). The hiM and hiI genes showed partially different patterns of overlap with these 5 modules of coexpressed genes during DG development. While hiM genes significantly overlap with turquoise, blue, and yellow modules, hiI genes significantly overlap with only turquoise module (Supplementary Figure 4b). Additionally, we performed pathway analyses on these 5 modules in BaseSpace (Supplementary Figure 4c). The turquoise module includes biogroups associated with the nucleus (e.g., "cell division", "Genes involved in Cell Cycle", and "mitosis"), and

blue module includes biogroups associated with synapse (e.g., “dendritic spine” and “synapse”). Similar biogroups were found in results of pathway analysis in Table1 (for example, biogroups such as “synaptic transmission” and “synapse” were involved in the results of pathway analysis for hiM genes; biogroups such as “Genes involved in Cell Cycle”, “mitosis”, and “cell division” were involved in the results of pathway analysis for hiI genes).

All information on the datasets and gene lists used in this study are provide as Supplementary Data 1 to 12.

Supplementary Methods

Computing overlap *P*-values of gene expression patterns in different datasets

BaseSpace can be used to compare the signatures in publicly available microarray datasets with a signature provided by the user using a "Running Fisher" algorithm, as previously described[1–5]. To enable comparison across different arrays, orthologs were identified for each pair of organisms. Ortholog identification was based on information obtained from Mouse Genome Informatics (MGI) at Jackson Lab (http://www.informatics.jax.org), HomoloGene at NCBI (http://www.ncbi.nlm.nih.gov), and Ensembl (http://www.ensembl.org). The overlap *P*-value, i.e., the direction of the correlation between two given gene signature sets (*b1*, *b2*), and the *P*-values between subsets of gene signatures are calculated as follows.

Each gene signature set was rank-ordered according to the absolute fold-change value. Upregulated and downregulated genes were denoted by positive and negative signs, respectively, to indicate directionality. A directional subset was generated for each direction, such as *b1+*, *b1-*, *b2+*, and *b2-*.

Next, all of the subset pairs were identified as *b1Di*, *b2Dj*, where *Di* and *Dj* were the available directions (+ or −) in *b1* and *b2*, respectively. The Running Fisher algorithm was applied to each subset pair. The top ranking genes in the first subset *b1Di* were collected as a group, *G*, and the second subset *b2Dj* was scanned from top to bottom in rank order to identify each rank with a gene matching a member in group *G*. At each matching rank, *K*, the scanned portion of the second subset *b2Dj,* consisted of *N* genes, and the overlap between group *G* and these *N* genes was defined as *M*. Fisher's exact test was performed at rank *K* to evaluate the statistical significance of observing *M* overlaps between a set of size *G* and a set of size *N*, where the set of size *G* comes from platform *P1,* and the set of size *N* comes from platform *P2*, given the sizes of *P1* and *P2* as well as the overlap between *P1* and *P2*. At the end of the scan, the best *P*-value was retained, and a multiple-hypothesis-testing correction factor was applied. The negative log of the multiple-testing-corrected best *P*-value ($P_{b1Di \rightarrow b2Dj}$) was a score ($S_{b1Di \rightarrow b2Dj}$) for the subset pair. Here, the subscript $b1Di \rightarrow b2Dj$ indicates that *b*1*Di* was the first subset used to define the top genes *G,* and *b*2*Dj* was the second subset that was used for the scan.

$$S_{b1Di \rightarrow b2Dj} = -\ln P_{b1Di \rightarrow b2Dj} \qquad (1)$$

Next, the Running Fisher algorithm was performed in the reverse direction. The same procedure in this reverse direction produced another score ($S_{b2Dj \rightarrow b1Di}$) for the same subset pair. The two scores were averaged to represent the magnitude of the similarity between the two subsets.

$$S_{b1Dib2Dj} = \frac{S_{b1Di \rightarrow b2Dj} + S_{b2Dj \rightarrow b1Di}}{2} \quad (2)$$

The P value ($P_{b1Dib2Dj}$) between $b1Di$ and $b2Dj$ was calculated using the following equation:

$$P_{b1Dib2Dj} = exp\ (-S_{b1Dib2Dj}) \quad (3)$$

A positive sign was assigned to pairwise correlation scores (S_{b1+b2+} and S_{b1-b2-}) for a subset pair of the same direction ($b1+b2+$, $b1-b2-$), and a negative sign was assigned to pairwise correlation scores (S_{b1+b2-} and S_{b1-b2+}) for a subset pair of opposite directions ($b1+b2-$, $b1-b2+$). Then, the overall score (S_{b1b2}) between $b1$ and $b2$ was calculated from the correlation scores (S_{b1+b2+}, S_{b1-b2-}, S_{b1+b2-}, and S_{b1-b2+}) of subset pairs using the following equation:

$$S_{b1b2} = \frac{S_{b1+b1+} + S_{b1-b2-}}{2} - \frac{S_{b1+b1-} + S_{b1-b2+}}{2} \quad (4)$$

The sign of S_{b1b2} reflected whether the two signatures were positively or negatively correlated. The overall P-value (P_{b1b2}) between $b1$ and $b2$ was calculated using the following equation:

$$P_{b1b2} = exp\ (-|S_{b1b2}|) \quad (5)$$

This overall P-value is referred to as the "overlap P-value" between two gene expression patterns in this paper.

Prediction of the subcellular of proteins coded by genes

To predict the subcellular localizations of proteins coded by genes in each dataset, COMPARTMENTS (http://compartments.jensenlab.org)[6] was used. COMPARTMENTS is a web resource that integrates evidence on protein subcellular localization from manually curated literature, high-throughput screens, automatic text mining, and sequence-based prediction methods. For each gene queried, it provides a score reflecting the localization to multiple cellular compartments (e.g., the plasma membrane, nucleus, cytosol, and so on) based on aggregating data from prediction algorithms (e.g., PSORT and YLoc).

The WoLF PSORT program[7], a sequence-based protein localization predictor, was used for the prediction of protein subcellular localization in this study. PSORT predicts subcellular localization based on various sequence-derived features such as sorting signals, binding domains, and amino acid composition. All PSORT scores were sorted by the number of stars (Spsort) assigned to a sequence-based prediction, from 0 to 3 (e.g., Rreb1: nucleus = 3, cytosol = 2, and other = 0; Fam107a: nucleus = 2, cytosol = 2, and other = 0). Detailed information about PSORT and Spsort score have been

described previously[6,7]. In this study, the score of each gene was determined by the highest Spsort score among the subcellular compartment(s). If there were two or more compartments sharing the highest score, the score of each gene was determined by dividing the total number by the number of compartments with the highest score (e.g., Rreb1: nucleus = 1; Fam107a: nucleus = 0.5, cytosol = 0.5). The subcellular distribution patterns of hiM/hiI genes are expressed as the proportion of the integrated scores of the top 50 genes.

Principal component analysis

Principal component analysis (PCA) was performed to reveal the relationship between datasets of marker genes. PCA was performed for datasets composed of hiM/hiI genes at three different timepoints: day 1, day 3, and day 10 after treatment. From the comprehensive gene lists, the 500 genes with the largest fold changes were used for PCA. The two primary components in the results of PCA, which are denoted as principal component 1 (PC1) and PC2, correspond to the x-/y-axes on the graphs. For data processing and PCA, we used R for Mac OS X (The R Foundation for Statistical Computing).

Reference

1. Kupershmidt, I. *et al.* Ontology-Based Meta-Analysis of Global Collections of High-Throughput Public Data. *PLOS ONE* **5,** e13066 (2010).

2. Takao, K. & Miyakawa, T. Genomic responses in mouse models greatly mimic human inflammatory diseases. *PNAS* **112,** 1167–1172 (2015).

3. Ryan, S. D. *et al.* Isogenic Human iPSC Parkinson's Model Shows Nitrosative Stress-Induced Dysfunction in MEF2-PGC1α Transcription. *Cell* **155,** 1351–1364 (2013).

4. Hagihara, H., Ohira, K., Takao, K. & Miyakawa, T. Transcriptomic evidence for immaturity of the prefrontal cortex in patients with schizophrenia. *Molecular Brain* **7,** 41 (2014).

5. Murano, T., Koshimizu, H., Hagihara, H. & Miyakawa, T. Transcriptomic immaturity of the hippocampus and prefrontal cortex in patients with alcoholism. *Scientific Reports* **7,** srep44531 (2017).

6. Binder, J. X. *et al.* COMPARTMENTS: unification and visualization of protein subcellular localization evidence. *Database (Oxford)* **2014,** (2014).

7. Horton, P. *et al.* WoLF PSORT: protein localization predictor. *Nucleic Acids Res* **35,** W585–W587 (2007).
