# Supplementary material for: Transcriptomic immaturity inducible by neural hyperexcitation is shared by multiple neuropsychiatric disorders
Source: Commun Biol. 2019 Jan 22;2:32. doi: 10.1038/s42003-018-0277-2 (PMC6342824; doi:10.1038/s42003-018-0277-2)
Supplement: Supplementary file 2 — Description of Additional Supplementary Files [file 42003_2018_277_MOESM2_ESM.docx]

**Description of Additional Supplementary Files**

**File Name**: Supplementary Data 1

**Description**: Gene list of hiM/hiI genes in mouse and human.

**File Name**: Supplementary Data 2

**Description**: Detailed information on the 92 microarray datasets used in this study.

**File Name**: Supplementary Data 3

**Description**: The lists of hiM and hiI genes included in datasets from patients with amyotrophic lateral sclerosis (ALS).

**File Name**: Supplementary Data 4

**Description**: The lists of hiM and hiI genes included in datasets from patients with Alzheimer’s disease (ALZ).

**File Name**: Supplementary Data 5

**Description**: The lists of hiM and hiI genes included in datasets from patients with autism spectrum disorder (ASD).

**File Name**: Supplementary Data 6

**Description**: The lists of hiM and hiI genes included in datasets from patients with bipolar disorder (BPD).

**File Name**: Supplementary Data 7

**Description**: The lists of hiM and hiI genes included in datasets from patients with Huntington’s disease (HD).

**File Name**: Supplementary Data 8

**Description**: The lists of hiM and hiI genes included in datasets from patients with major depressive disorder (MDD).

**File Name**: Supplementary Data 9

**Description**: The lists of hiM and hiI genes included in datasets from patients with Parkinson’s disease (PD).

**File Name**: Supplementary Data 10

**Description**: The lists of hiM and hiI genes included in datasets from patients with schizophrenia (SCZ).

**File Name**: Supplementary Data 11

**Description**: The lists of hiI genes shared in all datasets in each disease categories.

**File Name**: Supplementary Data 12

**Description**: The lists of hiI genes shared in all datasets in each disease categories.
